# Detection of *TP53* and *PIK3CA* Mutations in Circulating Tumor DNA Using Next-Generation Sequencing in the Screening Process for Early Breast Cancer Diagnosis

**DOI:** 10.3390/jcm8081183

**Published:** 2019-08-07

**Authors:** Begona Jimenez Rodriguez, Gema Diaz Córdoba, Alicia Garrido Aranda, Martina Álvarez, Luis Vicioso, Casilda Llácer Pérez, Cristina Hernando, Begoña Bermejo, Ana Julve Parreño, Ana Lluch, Matthew B. Ryder, Frederick S. Jones, Johannes Fredebohm, Frank Holtrup, María Isabel Queipo-Ortuño, Emilio Alba

**Affiliations:** 1 Unidad de Gestión Clínica Intercentros de Oncología Medica, Hospitales Universitarios Regional y Virgen de la Victoria de Málaga, 29010 Málaga, Spain; 2 Instituto de Investigación Biomédica de Málaga (IBIMA)-CIMES-UMA, 29010 Málaga, Spain; 3 Radiology Department, Hospital Clínico Universitario Virgen de la Victoria de Málaga, 29010 Málaga, Spain; 4 Histopathology Department, Hospital Clínico Universitario Virgen de la Victoria de Málaga, 29010 Málaga, Spain; 5 Hospital Clínico de Valencia, Instituto de Investigación Sanitaria INCLIVA, 46010 Valencia, Spain; 6 Centro de Investigacion Biomedica en Red Cancer (CIBERONC), 28029 Madrid, Spain; 7 Hospital Clínico de Valencia, Universidad de Valencia, Instituto de Investigación Sanitaria INCLIVA, 46010 Valencia, Spain; 8 Sysmex Inostics, GmbH, 20251 Hamburg, Germany

**Keywords:** breast cancer, circulating tumor DNA (ctDNA), molecular profiling, liquid biopsy, early-stage cancer

## Abstract

Circulating tumor DNA (ctDNA) has emerged as a non-invasive “liquid biopsy” for early breast cancer diagnosis. We evaluated the suitability of ctDNA analysis in the diagnosis of early breast cancer after mammography findings, comparing *PIK3CA* and *TP53* mutations between tumor biopsies and pre-biopsy circulating DNA. Matched plasma and frozen fresh tissue biopsies from patients with Breast Imaging-Reporting and Data System (BIRADS) 4c/5 mammography findings and subsequent diagnosis of primary breast cancer were analyzed using NGS TruSeq Custom Amplicon Low Input Panel (Illumina) and plasma SafeSEQ (Sysmex Inostics). The same plasma and tumor mutations were observed in eight of 29 patients (27.6%) with four in *TP53* and five in *PIK3CA* mutations. Sequencing analysis also revealed four additional ctDNA mutations (three in *TP53* and one in *PIK3CA*) previously not identified in three patients tissue biopsy. One of these patients had mutations in both genes. Age, tumor grade and size, immunohistochemical (IHC) subtype, BIRADS category, and lymph node positivity were significantly associated with the detectability of these blood tumor-derived mutations. In conclusion, ctDNA analysis could be used in early breast cancer diagnosis, providing critical clinical information to improve patient diagnosis.

## 1. Introduction

Breast cancer (BC) is the most common cancer in females worldwide, the second most common cause of death from cancer among males and females (only preceded by lung cancer), and a leading cause of premature mortality from cancer as measured by average and total years of life lost [1]. GLOBOCAN estimated that there would be about 2.1 million newly diagnosed cases of female breast cancer in 2018, accounting for almost one in four cancer cases among women worldwide [2]. As earlier detection and treatment of this cancer can improve survival, great effort should be placed on improving screening methods [3].

The current standard of care for BC screening is mammography [4]. Radiologists use the BIRADS (Breast Imaging-Reporting and Data System) criteria to describe the probability of a mammographic finding to be a malignant tumor [5], although, currently, it is still necessary to perform a biopsy of the lesion to confirm the diagnosis. Tissue biopsies are invasive, costly, time-consuming, and in some cases not amenable for repetition. Moreover, in many cases, these biopsies do not reflect the intratumor heterogeneity, which may have important consequences for personalized-medicine approaches that commonly rely on single tumor-biopsy samples to portray tumor mutational landscapes [6].

To overcome the limitations of tissue biopsies, recent studies have shown that molecular driver alterations in tumor tissues can be detected by liquid biopsy for circulating tumor DNA (ctDNA) [7], which comprise small fragments of double-stranded DNA (160–180 base pairs) released in to the bloodstream from cells through the process of necrosis and apoptosis in tumor tissues [8]. Increased levels of cell-free DNA (cfDNA) in the blood are frequently observed in cancer patients [9]. In addition, ctDNA levels have been correlated with tumor burden [10,11]. Previous studies have shown that ctDNA analysis is a good tool to assess the tumor genomic landscape in metastatic breast cancer patients [10,12]. Moreover, Madic et al. reported that *TP53* mutations were detected in the plasma of metastatic triple negative BC patients [13]. Interestingly, it was demonstrated that mutation tracking in ctDNA of breast cancer patients can identify early breast cancer patients at high risk of relapse [14]. Consistent with the previous data, Riva et al. found that a slow decrease of ctDNA level during neoadjuvant chemotherapy was strongly associated with shorter survival [15].

With the advance of next-generation sequencing (NGS) technologies, mutation analysis has become feasible and effective for clinical application in breast cancer [16]. According to the Cancer Genome Atlas (TCGA), the two most frequently mutated genes in primary BC are tumor protein p53 (*TP53*) and phosphatidylinositol-4,5-bisphosphate 3-kinase catalytic subunit alpha (*PIK3CA*), which are generally conserved mutations between primary tumors and metastatic disease [17].

Digital-PCR (dPCR) has become widely used to detect plasma DNA mutations due to its high accuracy, sensitivity, and specificity. However, dPCR is limited to the detection of a relatively small number of hotspot target alleles for which primers and/or probes are specifically designed but not useful for identifying unreported novel mutations. In a previous study in patients with early-stage breast cancer, the ctDNA detection rate was low, presumably due to the limited number of gene and loci analyzed [18]. In contrast, targeted NGS like SafeSEQ has recently emerged as a new tool to increase the sensitivity of massively parallel sequencing system instruments for the identification of rare variants in plasma DNA. This sequencing strategy uses single-molecule barcoding before PCR amplification to reduce sequencing error and increase accuracy [19,20,21]. To our knowledge, SafeSEQ has so far been applied to plasma samples of metastatic colorectal cancer [22] and to gastrointestinal stromal tumors (GIST) patients [23].

Nevertheless, it is still uncertain whether the driver molecular alterations in ctDNA can be detected by plasma cfDNA NGS based assays in primary breast cancer patients at screening and how these are related to the tumor tissue mutation found in the same patient.

The aim of the present study was to investigate whether ctDNA analysis can be used in early breast cancer diagnosis, by sequencing plasma DNA taken before tumor biopsy in patients with mammographic findings. Then, we have compared *PIK3CA* and *TP53* mutations between tumor biopsies and pre-biopsy circulating DNA as well as we have explored clinicopathological variables that may affect the detectability of these tumor-derived mutations in blood.

## 2. Experimental Section

### 2.1. Study Design and Patient Population

The pilot study included 29 patients with BIRADS 4c/5 mammography findings and subsequent diagnosis of primary breast cancer recruited at Virgen de la Victoria Hospital Málaga and Clinic University Hospital in Valencia. Blood samples were collected immediately (less than an hour) before tissue biopsy and prior to receiving any type of treatment. The matched tumor tissues were collected through core needle biopsies of breast tumors, which were subsequently fresh-frozen. Immunohistochemical (IHC) analysis was performed to quantify expression of human epidermal growth factor receptor 2 (HER2), hormone receptors (HR) and Ki67. Estrogen receptor (ER) and progesterone receptor (PR) were considered positive if tumors had more than 1% nuclear-stained cells. HER2 staining was scored on a scale of 0 to 3+, according to the HercepTest guidelines [24]. HER2 status was considered positive when graded as 3+, while 0 to 1+ were negative and 2+ was an inconclusive result and silver in situ hybridization (SISH) was performed in those cases to confirm positivity. Hormone receptor positive tumor plus Ki 67 index of <14% was considered a luminal A tumor while >14% was considered luminal B. IHC breast cancer subtypes were defined using a combination of these IHC markers as follows: Luminal A (ER-positive and/or PR-positive, HER2-negative and Ki-67 < 14%), luminal B (ER-positive and/or PR-positive, HER2-negative and Ki-67 ≥ 14%), HER2 positive (ER negative, PR negative, HER2 positive), and triple negative (ER, PR, and HER2 negative).

The study was approved by the research ethics committees at Virgen de la Victoria Hospital and Clínico de Valencia Hospital, all patients provided written informed consent. Research was conducted according to Good Clinical Practice and the Declaration of Helsinki.

### 2.2. DNA Extraction

Tumor DNA was isolated from fresh frozen tissue samples using the DNeasy Blood and Tissue Kit (Qiagen, Valencia, CA, USA) following the manufacturer’s instructions. Plasma blood samples of 10 mL were collected in STRECK tubes immediately before the tissue biopsy. Within 2 h after collection, plasma was separated from whole blood samples through centrifugation for 10 min at 3000 rpm at room temperature and stored at −80 °C until further use. The plasma samples were defrosted and were centrifuged again for 10 min at 13,000 rpm at room temperature prior to DNA extraction to remove debris. Isolation of DNA from 2 mL of plasma was performed using QIAamp Circulating Nucleic Acid Kit (Qiagen) according to the manufacturer’s instructions and eluted in 140 μL of AVE buffer (Qiagen). The total amount of amplifiable human genomic DNA isolated from plasma samples was quantified using a modified version of human long interspersed element 1 (LINE-1) real-time PCR assay and reported as genome equivalents (GE) [25,26].

### 2.3. Targeted Sequencing

The NGS study on fresh frozen tissue samples was performed using a customized design of TruSeq Custom Amplicon Low Input Panel (Illumina), which includes the full region of the *PIK3CA* and *TP53* genes (the most commonly mutated genes in early-stage BC according to TCGA). Libraries were constructed using Tru-Seq reagents from Illumina according to the standard protocol provided by Illumina. All DNA samples were diluted to the same initial concentration (25 ng/μL). In order to artificially increase the genetic diversity, 10% DNA from phage PhiX was added to the library of genomic DNA before loading on the flow-cell. Exon regions were captured in a solution using the Agilent SureSelect v.5 kit according to the manufacturer’s instructions (Agilent, Santa Clara, CA, USA). Paired-end sequencing was performed using a HiSeq 2500 Genome Analyzer (Illumina, San Diego, CA, USA). The sequences were aligned to the human genome reference sequence (hg19) using the Eland algorithm of CASAVA 1.8 software (Illumina, San Diego, CA, USA). The chastity filter of the BaseCall software of Illumina was used to select sequence reads for subsequent analysis. The ELANDv2 algorithm of CASAVA 1.8 software (Illumina, San Diego, CA, USA) was then applied to identify point mutations and small insertions and deletions. Potential somatic mutations were filtered and manually curated.

### 2.4. Plasma DNA Sequencing (SafeSEQ)

Plasma sequencing was performed using Plasma SafeSEQ (Sysmex Inostics, Hamburg, Germany). SafeSEQ analysis was performed on up to 10,000 GE (~33 ng DNA) per sample. Briefly, human genomic DNA isolated from plasma samples was amplified (15 cycles) in 10 replicate PCR wells using primers containing unique identifier sequences (UIDs), which consisted of 14 random bases with an equal probability of A, C, T, and G, to allow for the distinction of each template molecule. The amplified reactions were purified using AMPure XP beads (Beckman Coulter) and eluted in 250 mL of Buffer EB (Qiagen). A fraction of purified PCR product was then amplified in the second round of PCR with universal primers. The PCR products were purified with AMPure XP and sequenced on an Illumina MiSeq instrument. High-quality sequence reads were selected based on quality scores, which were generated by the Illumina sequencing instrument to indicate the probability that an error was made in base calling. The template-specific portion of the reads was matched to reference sequences. Reads from a common template molecule were then grouped based on the UIDs that were incorporated as molecular barcodes. Variants calls from the SafeSEQ assay were considered real if ≥90% of the PCR fragments with the same UID contain an identical mutation. Wells with fewer than 200 UIDs as a result of poor amplification were excluded from analysis. Once the analysis was performed, the following criteria were followed to call a mutation: (1) Variant allele frequency (VAF) of at least 0.05% and (2). The mutation needed to be detected in at least two out of 10 replicate wells.

### 2.5. Statistical Analysis

The statistical analysis was performed with SPSS (19.0, SPSS Inc., Chicago, IL, USA). For association analyses between clinicopathological variables and PIK3CA and TP53 derived-mutations in blood, Spearmen correlation and Fisher’s exact test (for categorical variables) were used. The threshold for statistical significance was set at *p* < 0.05.

## 3. Results

### 3.1. Patient Characteristics

The clinicopathological features of the tumors of the enrolled breast cancer patients are shown in Table 1. The average age of the patients was 64 years (range 44–92 years). The 29 primary breast tumors examined included 24 (82.7%) invasive ductal carcinoma (CDI), two (6.9%) invasive lobular carcinoma (CLI), two (6.9%) papillary carcinomas and one tubular carcinoma (3.4%). Fourteen tumors (48.3%) showed diameters of less than 2 cm, while 15 (51.7%) showed diameters of more than 2 cm. Axillary lymph node metastasis was detected by core needle biopsy in nine patients (31.0%). Ten (34.5%) patients without lymph node metastasis had tumors with diameters of more than 2 cm. Immunohistochemistry was used to determine IHC subtypes of tumors as follows, 14 (48.3%) luminal A tumors, 11 (37.9%) luminal B tumors, three (10.3%) triple-negative tumors and one (3.4%) HER2-positive tumor. With respect to hormone receptor status, 25 (86.2%) of 29 BC patients were estrogen receptor (ER) positive and 17 (58.6%) of 29 patients were progesterone receptor (PR) negative.

Concerning the tumor grade, the majority of the patients presented grade 2 tumors (16 patients, 55%) while seven patients (24.5%) had grade 3 tumors and five (17%) patients had grade 1 tumors. The tumor grade was unknown in one patient (3.5%).

### 3.2. Analysis of PIK3CA and TP53 Mutations in Fresh Frozen Tissue Samples of Primary Breast

Mutations of the *PIK3CA* and *TP53* genes were analyzed in genomic DNA from fresh frozen tissue samples of 29 primary breast cancer using the Illumina platform and the TruSeq Custom Amplicon Low Input Panel. Samples were successfully sequenced, and data quality was high, probably because DNA was isolated from fresh tissue material and had not been degraded. A total of 34 somatic mutations were detected in the 29 primary breast cancer patients. Of the 29 patients, 19 (65.5%) had *PIK3CA* mutations, six (20.7%) had *TP53* mutations, and four (13.8%) had both *PIK3CA* and *TP53* mutations. One (3.4%) patient had two *PIK3CA* mutations. In summary, all tumor samples were found to harbor at least one mutation.

In total, *PIK3CA* somatic mutations were detected in 23 of 29 tumors (79.3%), including eight tumors with c.3140A > G (27.6%), five with c.1633G > A (17.2%), three with c.3140A > T (10.3%), three with c.1624G > A (10.34%), two with c.1637A > G (6.9%), one with c.3145G > C (3.4%) and one with both c.3129G > A (3.44%) and c.3130A > C (3.4%) mutations (Table 2). With respect to the *TP53* gene, mutations were detected in 10 of 29 tumors (34.5%), including two tumors with c.524G > A (6.9%) and eight tumors with c.637C > T (3.4%), c.398T > A (3.4%), c.743G > T (3.4%), c.1028_1029delAG (3.4%), c.842A > C (3.4%), c.832C > T (3.4%), c.734G > T (3.4%), and c.587G > C (3.4%), respectively (Table 2). The VAF of the mutated *PIK3CA* allele in these tumors was 22.4% (range 7.4–82.4%) and 18.0% (range 29.3–75.2%) for *TP53*.

The *PIK3CA* mutations were detected in 13 (92.8%) of 14 luminal A tumors, nine (81.8%) of 11 luminal B tumors (one harboring two different *PIK3CA* mutations), and one (33.3%) of three triple-negative tumors. The *TP53* mutations were found in three (21.4%) of 14 luminal A tumors, four (36.4%) of 11 luminal B tumors, two (66.7%) of three triple-negative and one (100%) of one Her2-positive tumor. Interestingly, the *TP53* mutations coexisted with *PIK3CA* mutations in two of three luminal A tumors and in two of four luminal B tumors.

### 3.3. PIK3CA and TP53 Mutations Detected in ctDNA

Plasma samples from 29 primary early stage breast cancer patients were analyzed by SafeSEQ. The analysis revealed the presence of 13 mutations in the ctDNA of 10 patients out of 29 (34%). There were six different *PIK3CA* mutations (c.3140A > T, c.3145G > C, c.1651C > A, c.3140A > G, c.1633G > A, c.1624G > A) and seven different *TP53* mutations (c.398T > A, c.641A > G, c.743G > T, c.587G > C, c.637C > T, c.659A > G, c.748C > T). The median VAF for *TP53* was 1.03% (range 0.09–20.56%) and 0.17% for *PIK3CA* (range 3.6–0.01%).

One patient with a grade 3 luminal B tumor had mutations on both genes, *TP53* (c.743G > T) and *PIK3CA* (c.3140A > T). One patient with a grade 2 luminal A tumor had two different *PIK3CA* mutations (c.1651C > A and c.1633G > A) and one patient with a grade 2 luminal A tumor had two different *TP53* mutations (c.641A > G and c.748C > T) (Table 3). The rest of *TP53* mutations were mostly detected in patients with luminal B tumors (three of six (50%)), followed by one (16.7%) patient with triple-negative tumor and two (33.3%) patients with luminal A tumors. With respect to *PIK3CA* mutations, were detected mostly in patients with luminal A tumors (three of five (60%)), while two (40%) patients with *PIK3CA* mutations had luminal B tumors.

### 3.4. Comparison of PIK3CA and TP53 Mutations Detection Results from Tumor Tissue and ctDNA

The same plasma and tumor somatic mutations were observed in eight (27.6%) of 29 patients. Five *PIK3CA* and four *TP53* mutations were detected in eight matched pairs of primary tumors and plasma samples 3 *TP53* (c.398T > A, c.587G > C, c.637C > T), 4 *PIK3CA* (c.3140A > G, c.1633G > A, C.1624G > A, C.3145G > C), and one patient with mutations in both genes (TP53 c.743G > T, PIK3CA c.3140A > T) (Table 4).

Moreover, base transition (purine-pyrimidine and pyrimidine-purine) was more frequent than transversions (pyrimidine-pyrimidine and purine-purine). All these concordant somatic mutations were pathogenic, eight of them were missense mutations and one was a nonsense mutation. *PIK3CA* concordant mutations were detected in three (21.4%) of 14 luminal A tumors, and two (18.2%) of 11 luminal B tumors, while *TP53* concordant mutations were found in three (27.3%) of 11 luminal B tumors, and one (33.3%) of three triple-negative tumors. Moreover, all patients that showed these concordant mutations had grade 2–3 invasive ductal carcinoma.

Interestingly, there were three *TP53* mutations (c.641A > G, c.659A > G, c.748C > T) and one *PIK3CA* mutation (c.1651C > A) detected in plasma ctDNA of three different patients that were not detected in the matched tissue sample, all of them found in grade 2 luminal A tumors.

### 3.5. Clinicopathological Variables Associated with the Detectability of Tumor-Derived PIK3CA and TP53 Mutations in Blood

We analyzed the association between the detectability of tumor *PIK3CA* and *TP53* mutations in ctDNA and patient clinicopathological variables (Table 5). The analysis indicated that the presence of ctDNA mutations was significantly associated with a lower age of the patients (*p* = 0.040), higher tumor size (*p* = 0.033) and tumor grade (*p* = 0.041), the presence of lymph node positivity (*p* < 0.001), a BIRADS category 5 (*p* = 0.004) and the IHC cancer subtype (*p* = 0.025).

## 4. Discussion

In this pilot study, we have demonstrated the possible implementation of plasma tumor DNA detection as noninvasive means for early breast cancer screening.

Data are currently limited about whether ctDNA analyses would be applicable to the screening process in early breast cancer detection, in part because the low tumor burden of early stage disease makes the detection of ctDNA difficult [18,20] and very low levels of plasma ctDNA are not normally detectable [25,27]. In our study and with the aim of improving this detection problem, the novel approach of SafeSEQ was employed. This technique is able to detect rare mutations with an allele frequency <0.001% [19].

In our population of 29 breast cancer screening patients, the NGS analysis of the tumor biopsies detected *PIK3CA* mutations in 79.3% (23/29) and TP53 in 34.5% (10/29) of primary breast cancer patients. While, one third (10/29, 34%) of the patients were found to have mutations in plasma samples. In total, 13 mutations were found, six (46.1%) different *PIK3CA* mutations and seven (53.8%) different *TP53* mutations. The VAF in ctDNA was low (0.05%–3.60%) with the exception of one case with 20.56% in a patient later found to have lung metastasis in the CT scan performed as part of the staging process.

Of the 13 plasma mutations, nine were concordant with the mutations found in matched tissue samples (five *PIK3CA* and four *TP53* mutations) in eight of 29 patients. Of these nine mutations, four PIK3CA mutations within the PI3Ka (p.E545K and p.E542K) and PI3Kc (p.H1047R and p.H1047L) have been previously reported as important hotspots in breast cancer [28,29,30,31]. These findings may highlight the potential role of ctDNA to capture the molecular abnormalities of the tumor at the very first stage of the disease, even before having a diagnosis.

On the other hand, in this study four additional mutation, three *TP53* (c.641A > G, c.659A > G, c.748C > T) and one *PIK3CA* (c.1651C > A) not found in the tissue were found in plasma of three different patients, which did not have any undetected metastases or additional undiagnosed tumors. All these *TP53* mutations have been reported in the catalog of somatic mutations in cancer (COSMIC) as driver mutations. This finding may be related to the capability of ctDNA to capture the tumor heterogeneity, which is indeed a limitation of tumor biopsies [32].

To date, a few applications of the liquid biopsies have been integrated into daily clinical practice, such as for molecular profiling of the tumor and monitoring resistance [33]. In the case of breast cancer, some studies have demonstrated its potential role in tracking therapeutic response and detection of relapse [14,34,35]. In a study with patients with more advanced locoregional disease, Riva et al. detected *TP53* plasma mutations in 27 of 36 triple-negative patients (75%) before neoadjuvant chemotherapy treatment using dPCR [13]. Likewise, in another study *PIK3CA* (p.H1047R, p.E545K, and p.E542K) mutations in ctDNA were found in 22% of 110 stage I-III BC patients [36].

Two recent studies have also investigated the potential of liquid biopsy for the early detection of different type of cancer. Cohen et al. applied a screening method combining mutation detection in ctDNA with circulating protein markers in 1005 patients with nonmetastatic, clinically detectable tumors across eight common tumor types (ovary, liver, stomach, pancreas, esophagus, colorectal, lung, and breast). The median sensitivity of this test among the eight cancer types evaluated was 70% and ranged from 98% in ovarian cancers to 33% in breast cancers. At this sensitivity, the specificity was >99%, only seven of the 812 individuals without known cancers scored positive [37]. Chan et al. used detection of Epstein–Barr Virus (EBV) DNA in plasma to screen for nasopharyngeal carcinoma in 20.174 Chinese patients. Overall, the sensitivity and specificity of this approach were 97.1% and 98.6%, respectively [38].

In addition, several clinicopathological variables have been found associated with *PIK3CA* and *TP53* plasma ctDNA mutation detectability in early breast cancer patients. We found a trend for a higher plasma ctDNA mutation burden in patients with clinical characteristics associated with more aggressive disease factors such as higher tumor grade (grade II-III), and tumor size, BIRADS category 5, the presence of positive lymph nodes and the IHC subtype present, being more frequently mutated in luminal A tumors followed by luminal B tumors. Similar results have been described in a previous study in primary breast cancer patients where clinicopathological features, such as N stage and hormone receptor status, were associated with the detectability of tumor-derived mutations in blood [39]. Then, this finding may help to explain the absence of ctDNA mutations for some patients in the early breast cancer screening.

Regarding clonal hematopoiesis, detectable clonal expansions most frequently involved somatic mutations in *DNMT3A*, *ASXL1*, and *TET2* genes [40]. Furthermore, recent studies demonstrated that the genes more commonly associated with clonal hematopoiesis of indeterminate potential (CHIP) were *DNMT3A*, *ASXL1*, *TET2*, *JAK2*, *SF3B1*, *CBL*, *GNAS*, and *IDH2* [41]. Interestingly, when healthy individuals were assessed for CHIP, no alterations in driver genes related to solid cancers were detected [42]. For these reasons, clonal hematopoiesis was not analyzed in our population, since *TP53* and *PIK3CA* are commonly mutated genes in breast cancer.

## 5. Conclusions

In conclusion, to our knowledge, this is the first pilot study done in patients at the breast cancer screening process before undergoing any invasive diagnostic procedure or treatment, which shows that ctDNA analysis could be used in early breast cancer diagnosis. This study has demonstrated that ctDNA may reflect the *PIK3CA* and *TP53* tumor-derived mutations present in very early breast cancer patients. Nevertheless, several clinicopathological variables can affect the detectability of these ctDNA mutations. Although our study population is small and larger-scale studies will be necessary to validate our results, we can conclude that early ctDNA testing can provide critical clinical information that may improve patient diagnosis.

## Figures and Tables

**Table 1:** Clinicopathological characteristics of primary breast cancer patients.

Diagnostic Age (years) Median (range)	64 years (44–92)
**Mammographic Tumor Size *n* (%)**	
<2 cm	15 (51.7)
2 to 5 cm	13 (44.8)
>5 cm	1 (3.4)
**Tumor type**	
CDI	24 (82.7)
CLI	2 (6.9)
Papilar carcinoma	2 (6.9)
Tubular carcinoma	1 (3.4)
**Tumor grade *n* (%)**	
I	5 (17.2)
II	16 (55.2)
III	7 (24.13)
Unknown	1 (3.4)
**Axilar lymph node**	
Positive	9 (31.3)
Negative	20 (68.9)
**Progesterone receptor *n* (%)**	
Positive	12 (41.4)
Negative	17 (58.6)
**Estrogen receptor *n* (%)**	
Positive	25 (86.20)
Negative	4 (13.79)
**HER2 status *n* (%)**	
Positive	1 (3.4)
Negative	28 (96.6)
**IHC subtype *n* (%)**	
Luminal A tumor	14 (48.3)
Luminal B tumor	11 (37.9)
HER2-positive tumor	1 (3.4)
Triple negative tumor	3 (10.3)
**BIRADS category**	
4c	14 (48.3)
5	15 (51.7)

IDC: Invasive ductal carcinoma; ILC: Invasive lobular carcinoma; HER2: Human epidermal growth factor receptor 2; IHC: Immunohistochemistry; BIRADS: Breast Imaging-Reporting and Data System.

**Table 2:** Pathogenic mutations of PIK3CA and TP53 genes in tumors samples of primary breast cancer patients.

Patient ID	Gene	HGVS	Protein	Chr	hg19	Tumor VAF (%)	COSMIC
2MS	*PIK3CA*	c.3140A > T	p.H1047L	3	178952085	31.2	COSM776
2MS	*TP53*	c.524G > A	p.R175H	17	7578406	58.3	COSM10648
7MS	*PIK3CA*	c.3140A > G	p.H1047R	3	178952085	31	COSM775
9MS	*TP53*	c.637C > T	p.R213Ter	17	7578212	46.9	COSM10654
10MS	*PIK3CA*	c.3140A > G	p.H1047R	3	178952085	17	COSM775
13MS	*TP53*	c.398T > A	p.M133K	17	7578532	75.2	COSM11781
14MS	*PIK3CA*	c.3140A > G	p.H1047R	3	178952085	25.3	COSM775
16MS	*PIK3CA*	c.3140A > T	p.H1047L	3	178952085	28.2	COSM776
16MS	*TP53*	c.743G > T	p.R248L	17	7577538	43.1	COSM6549
17MS	*PIK3CA*	c.3140A > G	p.H1047R	3	178952085	30.1	COSM775
19MS	*PIK3CA*	c.1624G > A	p.E542K	3	178936082	47.7	COSM760
22MS	*TP53*	c.1028_1029delAG	p.E343AfsTer3	17	7573997	73.5	COSM5752326
23MS	*PIK3CA*	c.1633G > A	p.E545K	3	178936091	30.6	COSM763
23MS	*TP53*	c.524G > A	p.R175H	17	7578406	68.9	COSM10648
30MS	*PIK3CA*	c.1633G > A	p.E545K	3	178936091	56.2	COSM763
31MS	*PIK3CA*	c.1633G > A	p.E545K	3	178936091	10.6	COSM763
32MS	*PIK3CA*	c.3140A > G	p.H1047R	3	178952085	12.7	COSM775
35MS	*PIK3CA*	c.1633G > A	p.E545K	3	178936091	28.9	COSM763
36MS	*PIK3CA*	c.3140A > T	p.H1047L	3	178952085	44.9	COSM776
40MS	*PIK3CA*	c.1624G > A	p.E542K	3	178936082	25.5	COSM760
41MS	*TP53*	c.832C > T	p.P278S	17	7577106	29.1	COSM10939
43MS	*TP53*	c.734G > T	p.G245V	17	7577547	45.3	COSM11196
44MS	*TP53*	c.587G > C	p.R196P	17	7578262	35.8	COSM43814
47MS	*PIK3CA*	c.3140A > G	p.H1047R	3	178952085	10.6	COSM775
50MS	*PIK3CA*	c.3129G > A	p.M1043I	3	178952074	33.4	COSM29313
50MS	*PIK3CA*	c.3130A > C	p.N1044H	3	178952075	33.4	-----
52MS	*PIK3CA*	c.1637A > G	p.Q546R	3	178936095	11.6	COSM12459
56MS	*PIK3CA*	c.1624G > A	p.E542K	3	178936082	16.9	COSM760
65MS	*PIK3CA*	c.3140A > G	p.H1047R	3	178952085	23.6	COSM775
67MS	*PIK3CA*	c.3145G > C	p.G1049R	3	178952090	82.4	COSM12597
67MS	*TP53*	c.842A > C	p.D281A	17	7577096	44.6	COSM11665
68MS	*PIK3CA*	c.3140A > G	p.H1047R	3	178952085	15.9	COSM775
79MS	*PIK3CA*	c.1637A > G	p.Q546R	3	178936095	7.4	COSM12459
80MS	*PIK3CA*	c.1633G > A	p.E545K	3	178936091	29.5	COSM763

HGVS: Human Genome Variation Society; Chr: Chromosome; COSMIC: Catalog of somatic mutations in cancer; Tumor VAF: Tumor variant allele frequency.

**Table 3:** TP53 and PIK3CA mutations in ctDNA from primary breast cancer patients.

Patient ID	Gene	HGVS	SafeSEQ VAF (%)	Protein	hg19	COSMIC
7MS	*PIK3CA*	c.3140A > G	0.14%	p.H1047R	178952085	COSM775
9MS	*TP53*	c.637C > T	0.91%	p.R213Ter	7578212	COSM10654
13MS	*TP53*	c.398T > A	20.56%	p.M133K	7578532	COSM11781
16MS	*PIK3CA*	c.3140A > T	3.60%	p.H1047L	178952085	COSM776
16MS	*TP53*	c.743G > T	2.11%	p.R248L	7577538	COSM6549
30MS	*PIK3CA*	c.1651C > A	0.17%	p.L551I	178936109	----
30MS	*PIK3CA*	c.1633G > A	0.05%	p.E545K	178936091	COSM763
10MS	*TP53*	c.641A > G	3.88%	p.H214R	7578208	COSM43687c
10MS	*TP53*	c.748C > T	0.09%	p.P250S	7577533	COSM43695c
31MS	*TP53*	c.659A > G	0.16%	p.Y220C	7578190	COSM10758c
40MS	*PIK3CA*	c.1624G > A	0.20%	p.E542K	178936082	COSM760
44MS	*TP53*	c.587G > C	1.03%	p.R196P	7578262	COSM43814
67MS	*PIK3CA*	c.3145G > C	0.39%	p.G1049R	178952090	COSM12597

HGVS: Human Genome Variation Society; COSMIC: Catalog of somatic mutations in cancer; SafeSEQ VAF: Plasma variant allele frequency.

**Table 4:** Concordant PIK3CA and TP53 somatic mutations between tumors and ctDNA in primary breast cancer patients.

Patient ID	Diagnostic Mammography	Markers	Type	Gene	HGVS	SafeSEQ VAF (%)	Protein	Tumor VAF (%)	COSMIC	Chr
13MS	IDC, grade 3	ER+/HER2+	Luminal B	*TP53*	c.398T > A	20.56	p.M133K	75.2	COSM11781	17
16MS	IDC, grade 3	ER+/HER2+	Luminal B	*PIK3CA*	c.3140A > T	3.60	p.H1047L	28.2	COSM776	3
16MS	IDC, grade 3	ER+/HER2+	Luminal B	*TP53*	c.743G > T	2.11	p.R248L	43.1	COSM6549	17
44MS	IDC, grade 3	ER+/HER2+	Luminal B	*TP53*	c.587G > C	1.03	p.R196P	35.8	COSM43814	17
9MS	IDC, grade 3	ER-/PR-/HER2-	Triple negative	*TP53*	c.637C > T	0.91	p.R213Ter	46.9	COSM10654	17
67MS	IDC, grade 2	ER+/HER2−	Luminal A	*PIK3CA*	c.3145G > C	0.39	p.G1049R	82.4	COSM12597	3
7MS	IDC, grade 2	ER+/HER2−	Luminal A	*PIK3CA*	c.3140A > G	0.14	p.H1047R	31	COSM775	3
30MS	IDC, grade 2	ER+/HER2−	Luminal A	*PIK3CA*	c.1633G > A	0.05	p.E545K	56.2	COSM763	3
40MS	IDC, grade 1	ER+/HER2+	Luminal B	*PIK3CA*	c.1624G > A	0.20	p.E542K	25.5	COSM760	3

IDC: Invasive ductal carcinoma; HGVS: Human Genome Variation Society; SafeSEQ VAF: Plasma variant allele frequency; Tumor VAF: Tumor variant allele frequency; COSMIC: Catalog of somatic mutations in cancer; Chr: Chromosome.

**Table 5:** Clinicopathological variables influencing ctDNA mutation. detection in early breast cancer patients.

Characteristics	Total	ctDNA-Positive ^b^	* *p* Value
	*n*	*n* (%)	
Diagnostic age (years)			0.040
<36	9	7 (77.8)	
36–50	10	4 (40)	
>66	10	2 (20)	
Tumor size			0.033
<2 cm	15	2 (13.3)	
2 to 5 cm	13	10 (76.9)	
>5 cm	1	1 (10)	
Tumor grade			0.041
I	5	1 (20)	
II	16	6 (37.5)	
III	7	6 (85.7)	
Unknown	1	0 (0)	
Axilar lymph node			<0.001
Positive	9	9 (100)	
Negative	20	4 (20)	
Progesterone receptor			0.615
Positive	12	5 (41.7)	
Negative	17	8 (47)	
Estrogen receptor			0.571
Positive	25	12 (48)	
Negative	4	1 (25)	
HER2 status			0.655
Positive	1	1 (10)	
Negative	28	12 (42.8)	
IHC subtype			0.033
Luminal A tumor	14	7 (50)	
Luminal B tumor	11	5 (45.4)	
HER2-positive tumor	1	0 (0)	
Triple-negative	3	1 (33.3)	
			
BIRADS category			0.004
4	14	1 (7.1)	
5c	15	12 (80)	
			

^b^ ctDNA was defined as positive if tumor-derived mutations for TP53 and PIK3CA could be detected in blood. * *p*-value < 0.05 represents statistical significance.

## Abbreviations

BC	Breast cancer;
BIRADS	Breast Imaging-Reporting and Data System;
ctDNA	Circulating tumor DNA;
NGS	Next generation sequencing;
TCGA	Cancer Genome;
TP53	tumor protein p53;
PIK3CA	phosphatidylinositol-4;5-bisphosphate 3-kinase catalytic subunit alpha;
dPCR	Digital-PCR;
GIST	Gastrointestinal stromal tumors;
IHC	Immunohistochemical;
HER2	human epidermal growth factor receptor 2;
HR	Hormone receptors;
ER	Estrogen receptor;
PR	Progesterone receptor;
SISH	Silver in situ hybridization;
VAF	Variant allele frequency;
CDI	Invasive ductal carcinoma;
CLI	Invasive lobular carcinoma;
EBV	Epstein–Barr Virus;
OR	Odds ratio;
CI	Confidence interval;
COSMIC	Catalog of somatic mutations in cancer;
CHIP	Clonal hematopoiesis of indeterminate potential
